# The Trojan Horse Model in *Paracoccidioides*: A Fantastic Pathway to Survive Infecting Human Cells

**DOI:** 10.3389/fcimb.2020.605679

**Published:** 2021-02-11

**Authors:** Gustavo Giusiano

**Affiliations:** Mycology Department, Instituto de Medicina Regional, Universidad Nacional del Nordeste, Consejo Nacional de Investigaciones Científicas y Técnicas (CONICET), Resistencia, Argentina

**Keywords:** dissemination, transmigration, internalized parasitic cells, Paracoccidioidomycosis, immune response evasion

## Abstract

Paracoccidioidomycosis (PCM) is the most relevant systemic endemic mycosis limited to Latin American countries. The etiological agents are thermally dimorphic species of the genus *Paracoccidioides*. Infection occurs *via* respiratory tract by inhalation of propagules from the environmental (saprophytic) phase. In the lung alveoli the fungus converts to the characteristic yeast phase (parasitic) where interact with extracellular matrix proteins, epithelial cells, and the host cellular immunity. The response involves phagocytic cells recognition but intracellular *Paracoccidioides* have demonstrated the ability to survive and also multiply inside the neutrophils, macrophages, giant cells, and dendritic cells. Persistence of *Paracoccidioides* as facultative intracellular pathogen is important in terms of the fungal load but also regarding to the possibility to disseminate penetrating other tissues even protected by the phagocytes. This strategy to invade other organs *via* transmigration of infected phagocytes is called Trojan horse mechanism and it was also described for other fungi and considered a factor of pathogenicity. This mini review comprises a literature revision of the spectrum of tools and mechanisms displayed by *Paracoccidioides* to overcame phagocytosis, discusses the Trojan horse model and the immunological context in proven models or the possibility that *Paracoccidioides* apply this tool for dissemination to other tissues.

## Introduction

Onygenalean (Ascomycota) organisms including *Paracoccidioides*, have typically adapted to saprobic conditions in soil but also to the live tissues of animal hosts. This biotrophic lifestyle is possible thanks to genomics adaptations allowing them the capability to degrade animal substrates suggesting a duality in lifestyle that could enable pathogenic species of Onygenales to transfer from soil to animal hosts (Desjardins et al., 2011). The potential of this thermodimorphic fungi to become a pathogen and to invade a host it’s based on numerous fungal strategies to escape and to bypass the host defense mechanisms (Teixeira et al., 2014; De Oliveira et al., 2015; Camacho and Niño-Vega, 2017).

*Paracoccidioides* species complex is widely distributed on Latin American soils with high incidence in South America (Negroni, 1993; Restrepo et al., 2012). Paracoccidioidomycosis (PCM) process start after inhalation of the environmental morphotype, when reaches the lung alveoli. At this point, the dimorphic transition to the yeast form and the interaction with the extracellular matrix (ECM) proteins, epithelial cells, and the host cellular immunity mediated by the phagocytic cells of the innate immune and adaptive systems, they are the first steps in a complex relationship between *Paracoccidioides* and the host that can lead to a granulomatous disease. This multi-factorial host–pathogen interactions involves fungal virulence factors, adaptation, adhesion and invasion depending on the host immune status and its response (Negroni, 1993; González et al., 2005; González et al., 2008a; De Oliveira et al., 2015; Hernández-Chávez et al., 2017).

In this damage-response framework, the host attempt to kill the infecting microbe causing none or the minimum possible damage. On the other hand, *Paracoccidioides* spp. develops several tools as strategies to evade the host immune response (González and Hernández, 2016; Camacho and Niño-Vega, 2017). One of the most interesting mechanism is the ability to survive inside the phagocytes as a facultative intracellular pathogen (Brummer et al., 1989; Moscardi-Bacchi et al., 1994). This strategy could allow *Paracoccidioides* to leave the lung and to penetrate other tissues protected by the phagocytic cells (Silvana dos Santos et al., 2011). This important mechanism of pathogenesis, involving carriage inside the infected macrophage or dendritic cell, allowing extrapulmonary dissemination phagocytes associated, is named Trojan horse model.

### Phagocytes Activation

Phagocytosis followed by degradation of the fungal particles internalized by phagocytic cells is an essential innate immune response to prevent the dissemination. Initially, the response involves neutrophils, alveolar macrophages, and dendritic cells (DCs) recognition. Their digestive and killing capabilities will be decisive to the destiny of the infectious process, then they will stimulate the adaptive immune system through their cytokines and chemokines. All phagocytes exist in degrees of readiness. During an infection, they receive chemical signals which prepares for its specific function. Resistance against *Paracoccidioides* infection depends mainly on the phagocytes being activated, which exhibit an increased capacity to ingest and fungicidal functions. Such events are modulated by fungal components and host factors. Therefore, activation of these cells is essential (Cano et al., 1998; Rodrigues et al., 2007; Thind et al., 2015; González and Hernández, 2016; Marcos et al., 2016; Camacho and Niño-Vega, 2017).

The recognition of fungal wall components named pathogen-associated molecular patterns (PAMPs) by pathogen recognition receptors (PRRs) initiates the complex host innate immune response. These conserved transmembrane or intracytoplasmatic PRRs include the Toll-like receptors (TLRs), mannose receptors (MR), complement receptors (CR), and the family of C-type lectin receptors (CLRs) such as CRL dectin-1, 2, and 3, among others. This interaction drive to the activation of the innate immune system cells and the succeeding production of mediators involved into the removal of the agent and to the control of the adaptative immune responses (Calich et al., 2008; Loures et al., 2014; Loures et al., 2015; Preite et al., 2018).

Knowledge about the immunopathogenesis of PCM is based on *in vivo* and *in vitro* experimental studies (González et al., 2008b; González and Hernández, 2016). Human and murine models showed de crucial role of TLRs inducing the production of inflammatory cytokines that drives naive T cells to Th and Treg cells. Patients with T cell deficiencies are more susceptible to fungal infections such as PCM. T cells are the major source of cytokines and lead to generate Th1 cytokines in order to activate macrophages and DCs in a next step. Th1 cells secrete interferon gamma (IFN-γ) and tumor necrosis factor (TNF-α), both cytokines activates macrophages and DCs enhancing their ability to kill or inhibit intracellular fungi and to present antigens to T lymphocytes (Cano et al., 1998; González et al., 2003; Silvana dos Santos et al., 2011; Thind et al., 2015; Marcos et al., 2016; Camacho and Niño-Vega, 2017).

The cytokine balance limited by the mutual regulation between Th1, Th2, Th17, and Treg polarization is necessary in order to optimize clearance and minimize inflammatory damage to the infected tissues. There are two possible outcomes of this balance that can result in control and removal of the fungal infection or lead to persistence of the infection and progress to a severe pathology (Olszewski et al., 2010; de Castro et al., 2013). Th1 and Th2 patterns of cytokine expression have been associated with PCM resistance and susceptibility, respectively (Cano et al., 1998; Mamoni et al., 2002; Benard, 2008; de Castro et al., 2013).

### How to Survive and Even Multiply Into the Phagocytes

**Figure 1:**
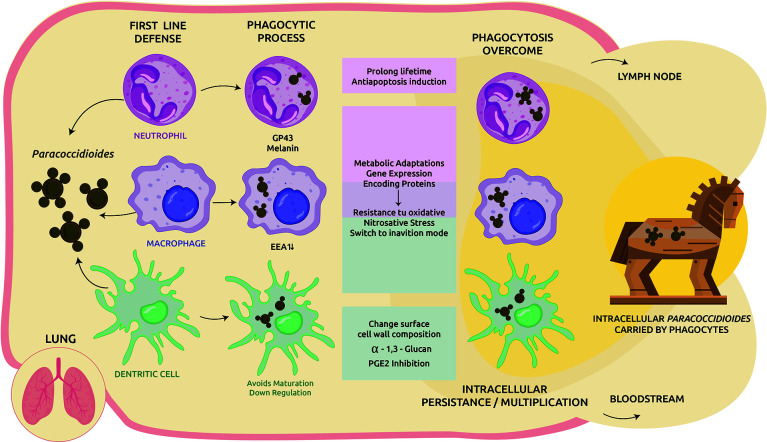
Cellular response to *Paracoccidioides* infection in the lung and immune-evasion mechanisms. Upon inhalation the saprophytic form converts to the yeast-like parasitic and trigger the host cellular immune response. Phagocytes are motivated to clear the invasive fungi. In addition to its own structural virulence determinants such as gp43, melanin, among others, *Paracoccidioides* spp. applies several strategies to overcome the host harsh environment, including: modulate host apoptosis, metabolic adaptations, and expression of genes to achieve an inanition mode and also resistance to the host oxidative burst. When intracellular survival is possible, part of the phagocytosed fungi could be transported by DCs/macrophages to lymphoid tissues or other organs *via* circulation as facultative intracellular pathogens but protected by phagocytes (Trojan horse mechanism).

The phagosome has a powerful antimicrobial effect. A combination of factors gives this organelle sufficient capability to eliminate pathogens, from inducing nutrients and trace elements deficiencies to producing different antimicrobial compounds that stress the internalized microbe (González and Hernández, 2016). Several studies trying to elucidate how the parasitic yeast-like form of *Paracoccidioides* manage to survive inside phagocytic cells. The strategy to evade the hostile host conditions includes a multiplex approach (**Figure 1**).

### Polymorphonuclear Neutrophils

They are the most abundant leukocytes and the main effector cells in the prevention of fungal infections. Polymorphonuclear neutrophils (PMNs), the primary phagocytic cells of the innate immune system, when activated *via* TLRs initiate the inflammatory response against *Paracoccidioides*. Chemokines produced by neutrophils are involved in the chemotaxis for the rapid migration of immune cells to the infection site. Neutrophils granules contain antimicrobial peptides, nucleolytic enzymes, and also oxygen metabolites acting in the removal process disrupting the cell membrane of fungus (Traynor and Huffnagle, 2001; Rodrigues et al., 2007; González et al., 2008b; Pathakumari et al., 2020). In addition, PMNs are able to produce extracellular traps (NETs), these structures are able to capture microbials, degrade their virulence factors, and eliminate the pathogens (Mejía et al., 2015; Restrepo et al., 2015).

The balance between pro-inflammatory and anti-inflammatory cytokines is a prerequisite for a successful host/fungal interaction. Participation of TLR2, TLR-4, and dectin-1 receptors in recognition, internalization, and consequent activation of neutrophils was demonstrated in human neutrophils stimulated by *Paracoccidioides brasiliensis*. In this study, the more virulent strain induced production of only TNF-α. The less virulent, in contrast, triggers a controlled immune response with balanced production of TNF-α and IL-10, preferentially recognized by TLR2 and dectin-1 (Bonfim et al., 2009).

Non activated cells failing to exhibit an antifungal activity was demonstrated. Due to the capability of human PMNs to release higher oxygen metabolites, an activation process by IFN-γ, TNF-α, and GM-CSF cytokines is required for killing *P. brasiliensis* (Rodrigues et al., 2007). This fact is important to understand one of the mechanisms through which *Paracoccidioides* could adapt to the host environment and survive. Transcriptome analysis of *P. brasiliensis* reveals many resources of this fungus as antioxidant defense system to combat reactive species. The parasite’s abilities to overcame the oxidative and nitrosative stress by genes coding proteins involved in this response were described and include catalase and superoxide dismutase isoenzymes, peroxiredoxin, cytochrome c peroxidase, among others (Campos et al., 2005).

At this point, the cytokine balance is also critical. The lack of an adequate *in vivo* activation of PMNs as a consequence of a depressed Th1 response releasing low levels of cytokines, leads to the possible inability of PMNs to successfully kill *Paracoccidioides* (Kurita et al., 1999; Rodrigues et al., 2007).

PMNs ingest yeast cells of *P. brasiliensis* through a typical phagocytic process. These phagocytes are shortlived cells, after few hours they undergo spontaneous apoptosis. Intracellular microorganisms may block or delay this process to create an environmental for their survival and replication. *In vitro* studies showed that *P. brasiliensis* can prolong the lifetime of normal PMNs and also induce an anti-apoptotic process associated with an increase in PMNs IL-8 production as an strategy to facilitate intracellular persistence (Acorci et al., 2009).

For decades, the role of PMNs in some granulomatous diseases they have been studied showing an abnormal function with a significantly lower ability to digest *P. brasiliensis in vitro* than PMNs from normal individuals or from patients with unrelated diseases (Goihman-Yahr et al., 1980).

This mechanism is another pathway that contribute to understand the PCM pathogenesis. Inhibition of phagocytic cells apoptosis allow *P. brasiliensis* to survive within the PMNs, gain time for multiplication and also dissemination.

### Macrophages

Lung macrophages participate as one of the main mechanisms of cellular immunity trying to prevent the parasitic invasion of host tissues and its dissemination through phagocytosis or granuloma formation (González et al., 2008b). Studies using murine macrophages and also proteomic analysis showed the activations process as a requirement to obtain a more vigorous defense with significantly more capability to kill the yeast-like phase of *P. brasiliensis*. Otherwise, the ingested *P. brasiliensis* can multiply inside non-activated cells (Brummer et al., 1989; Cano et al., 1992; Moscardi-Bacchi et al., 1994; Parise-Fortes et al., 2000; Chaves et al., 2019).

T cells are mandatory for antifungal host defense. Th1 cells are involved in cell-mediated immunity supporting classical activation of macrophages for fungal clearance and are associated with strong proinflammatory responses. In contrast to the protective role of Th1, a Th2 humoral with insufficient production or deficient IFN-γ, TNF-α, and IL-12 response, is non-protective and was related with fungal persistence and pathology. Th1 pattern is associated to asymptomatic and mild PCM forms while an Th2 pattern has been related with progressive juvenile and multifocal forms (Cano et al., 1998; Mamoni et al., 2002; González et al., 2003; González et al., 2008b; Olszewski et al., 2010; de Castro et al., 2013). A strong Th2 response suppresses the Th1 and Th17 response and triggers the alternative macrophage activation mediated by IL-4 and IL-13. The imbalance of Th2 responses, is inadequate to control the infection and lead to an uncontrolled inflammatory host response. This pathway do not express the fungicidal effect of the nitric oxide and other intermediates and has been associated with intracellular *Paracoccidioides* survival since it is not affected by the nitrosative stress (González et al., 2008b; Olszewski et al., 2010; Borghi et al., 2014). Th2 response is also characterized by IgG4 and IgE, low macrophages activation, granulomas, and eosinophilic inflammation (Mamoni et al., 2002).

TLRs shows their important participation in the effector and regulatory mechanisms of innate and adaptative immunity against fungal infections. Even if TLRs receptors promote an immune response against infectious agents, experimental models demonstrated that parasitic phase of *Paracoccidioides* could use not conventional phagocytic receptors such as TLR2 and TLR4 to penetrate into macrophages and infect mammalian hosts. Although this process should generate a phagocytic process, the killing activity was demonstrated not able to reduce the fungal burden. *P. brasiliensis* seems to use TLRs as a virulence mechanism, which facilitates its access into murine macrophages *in vitro* and *in vivo*. Despite their TLR-mediated activation, macrophages are not able to control fungal growth. However, the interaction between TLR and other PRRs can result in different effector (Th1, Th2, and Th17) and regulatory responses (Treg), which ultimately determine disease outcome. The recognition of *P. brasiliensis via* host TLR2 and TLR4 receptors of innate immunity is considered an escape mechanism that allows the fungus to survive and replicate inside macrophages (Calich et al., 2008).

Microbicidal activity of macrophages include the induction of a low availability of nutrients but also, they activate an oxidative burst. At this point, *Paracoccidioides* display a spectrum of tools to adapt into in the intracellular environment, requiring metabolic adaptations.

A decisive success is based on its resistance to oxidative and nitrosative stresses and glucose deprivation. Reactive oxygen species (ROS) and reactive nitrogen species (RNS) are generated inside the phagolysosome, such us nitric oxide, peroxynitrite, superoxide anion radical, and hydroxyl radical. In the face of the oxidative and nitrosative stress, *Paracoccidioides* triggers a powerful antioxidant defense system expressing several enzymes including catalases, superoxide dismutases, thioredoxin, and particularly cytochrome c peroxidase (González et al., 2000; de Arruda Grossklaus et al., 2013; Parente-Rocha et al., 2015; Marcos et al., 2016; Bueno et al., 2016; Chaves et al., 2019). The central role of the alternative oxidase (PbAOX) in the intracellular redox balancing and in the resistance of *P. brasiliensis* to the oxidative burst created by alveolar macrophages was also demonstrated (Hernández Ruiz et al., 2011).

Adaptation, in order to survive under this stress, also includes metabolic changes such us an alternative metabolic pathway during carbon starvation. Several studies including proteomic and transcriptomic analysis showed the shift of *P. brasiliensis* to an “inanition mode,” including an increase in the synthesis of glucose by gluconeogenesis and ethanol production, amino acid degradation and utilization of fatty acids by beta-oxidation (Lima et al., 2014; Parente-Rocha et al., 2015; Chaves et al., 2019). Metabolic alterations also include the activation of the pentose phosphate pathway to provide NADPH, a reducer substrate used to reduce the oxidative effects when exposed to peroxide hydrogen (de Arruda Grossklaus et al., 2013).

Many fungal genes have been studied as probably involved in the survival of *P. brasiliensis* in the host. Genes encoding proteins essential to the life and those indispensable for the interaction with the host were reported. Using murine macrophages transcriptional plasticity of *P. brasiliensis* in response to the hostile macrophage intracellular environment was reported. To adapt and consequent survive, *P. brasiliensis* expresses genes associated with glucose and amino acid limitation, cell wall construction and oxidative stress (Popi et al., 2002). It has also been demonstrated that *Paracoccidioides* could develop a fermentation process to obtain energy enabling its adaptation to glucose-poor microenvironments. Even more, can also produce ATP under low oxygen conditions, in turn reducing the reactive oxygen species levels produced by the host (Tavares et al., 2015).

In addition, lung murine infection models showed that *Paracoccidioides* increased the expression of serine proteinase. This protein is involved in cell rescue, defense, and as a virulence factor that favors survival upon nitrogen deprivation, as well as tissue invasion (Parente et al., 2010; Lacerda Pigosso et al., 2017). Increased expression of heat shock proteins and proteins involved in detoxification and stress response were observed using proteomic analysis in *P. brasiliensis* recovered of primed and non-primed macrophages (Chaves et al., 2019).

On the other hand, host cells try to prevent intracellular survival and multiplication sequestering essential fungal nutrients such as iron and zinc using high-affinity proteins, transferrin, and ferritin. Iron is required for the saprophytic phase-to-yeast transition, necessary for the pathogenic process development, as well as yeast replication inside macrophages and monocytes (González et al., 2007). In order to persist inside this environmental condition, *Paracoccidioides* activate effectives iron and zinc uptake pathways, adjusting their energy metabolism to an iron-independent mode by increasing glycolytic activity and also expression of genes involved in the production of siderophores (Parente et al., 2011; Silva-Bailão et al., 2014). Even more, develops a non-traditional reductive iron assimilation pathway, transporting zinc and iron inside the fungal cell *via* iron reduction and zinc-regulated transporter homologs (Zrt1 and Zrt2) (Camacho and Niño-Vega, 2017). Another iron acquisition mechanism mediate by the putative hemoglobin receptor Rbt5 was demonstrated. *Paracoccidioides* Rbt5 was able to bind to hemin, protoporphyrin, and hemoglobin *in vitro* and could function as a heme group receptor, which could help in the acquisition of iron from host sources (Bailão et al., 2014).

Gp43 is the *Paracoccidioides* surface main antigen. This high mannose glycoprotein of 43 kDa is an adhesin, important as one of the mediators of fungus adhesion to host epithelial cells and macrophages internalization. In peritoneal macrophages from resistant and susceptible mice, gp43 acts an inhibitor of phagocytosis and the intracellular fungal killing, even induce protection. Therefore, is considered as one of the evasion mechanisms of the primary infection in susceptible hosts and to establish the fungal infection in distant niches favoring the dissemination (Popi et al., 2002; Konno et al., 2012; De Oliveira et al., 2015; Camacho and Niño-Vega, 2017). Gp43 also prevents the release of nitric oxide from macrophages reducing the nitrosative stress and stimulates IL-10 liberation, reducing the inducible nitric oxide synthase expression and its enzymatic activity. The suppressor effect of IL-10 blocks the IFN-γ and TNF-α-induced activation of phagocytic cells, by inhibiting their fungicidal activity and ability to produce the oxidative metabolites (oxide nitric and oxygen peroxide) involved in fungus killing. Gp43 mediates another escape mechanism of *Paracoccidioides*, impairing the ingestion process and the interaction macrophage–fungus, inducing the deactivation of the phagocytic cell (Popi et al., 2002; Moreira et al., 2010). In addition, the early monocyte/macrophage secretion of IL-10, particularly when these cells were challenged with gp43 was observed. In patients with both the acute/subacute and chronic forms of PCM, the imbalance in cytokine production was involved in the gp43-hyporesponsiveness and a marked (non-protective) antibody production. (Benard et al., 2001).

*Paracoccidioides* produce cell wall-associated melanin-like components *in vivo* and during infection. Melanin is another virulence factor that has been shown to interfere with host defense mechanisms enhancing the resistance to immune effector cells attacks (Taborda et al., 2008). In macrophage-like cell lines, the phagocytic index for melanized *P. brasiliensis* yeast cells was half that for the non-melanized cells. Yeast melanization interfere the binding of macrophages lectin receptors to cell wall components, consequently they are poorly phagocytized and more resistant to the antifungal activity of murine macrophages (da Silva et al., 2006).

One more survival strategy used when infected macrophages are established consists in the inhibition of the phagosome-endosome fusion. *Paracoccidioides* decrease the expression of the endocytic protein EEA1 (early endosome antigen 1) that has a critical function as organelle-tethering molecule responsible for traffic endosomal. Therefore, cellular nutrition is impaired and also the traffic of *Paracoccidioides* yeast for it final destruction in the lysosome (Voltan et al., 2013).

### Dendritic Cells

Lung cells such as DCs are part of the first line of defense against *Paracoccidioides*. DCs, as antigen-presenting cells, also plays a crucial role as sentinels in peripheral tissues inducing cell-mediated immune responses. PAMP-dependent or independent activation is also required. They capture antigens, processed, and converted these proteins to peptides that are immediately presented on major histocompatibility complex molecules recognized by T lymphocytes. DCs migrates to the lymph nodes, present antigens and initiate T cell activation/responses. These phagocytic are involved in detection, binding, phagocytosis, processing, antigen presentation, T cell activation and killing of the organism (Cano et al., 1998; Silvana dos Santos et al., 2011; Thind et al., 2015; Marcos et al., 2016; Camacho and Niño-Vega, 2017).

To adapt for survival in adverse conditions or stress, fungus has the ability to modify its cell wall structure and also composition. Polysaccharides of the cell wall are the main fungal PAMPs and trigger the immune response when are recognized by PRRs. Nevertheless, *Paracoccidioides* display strategies to evade recognition by phagocytic cells, changing the amount of certain surface cell wall components (Hernández-Chávez et al., 2017). During the morphologic change, cell wall composition of dimorphic fungi is altered as well as the carbohydrate polymer structure. Filamentous phase contains both β- and α-(1,3)-glucans, but conversion to the parasitic yeast form produce an increase of the much less immunogenic α-(1,3)-glucan (Marcos et al., 2016). Several studies demonstrated that DCs maturation is altered by the parasitic form, influencing the susceptibility to this fungus. When monocytes migrate to the infection site, they interact with components of *Paracoccidioides* cell wall. In this sense, the critical role of its cell wall in the host immune response during PCM was postulated. Two cell wall fractions, one constituted mainly by α-glucan and other by β-(1,3)-glucans, chitin, and proteins and the alkali-soluble were investigated, demonstrating the induction of a dysregulation in DCs differentiation. *Paracoccidioides* cell wall α-glucan, presented as the mayor neutral polysaccharide in the yeast phase, also influences favoring Th2 polarization and contributes to pathogen persistence (Puccia et al., 2011; Souza et al., 2019). On the other hand, the lower efficiency of DCs from mice susceptible to *P. brasiliensis* in inducing a Th1 response was observed, an effect that could be related to the progression of the disease *in vivo* (Almeida and Lopes, 2001).

Other *in vitro* studies using human immature DCs also demonstrated that *P. brasiliensis* inhibit prostaglandin E2 production by DCs, impairing its maturation in response to this fungus and showing another evasion mechanism. These authors suggest opposite mechanisms applied by *P. brasiliensis* to scape DCs and monocytes responses, since increased production of PGE2 by monocytes inhibits their killing mechanism, while inhibited production by DCs avoid their maturation (Fernandes et al., 2015).

The main immunodominant glycoprotein gp43 was reported affecting many functions of the host phagocytic cells and might be used by *Paracoccidioides* to reduce the effectiveness of the immune response. Studies with *P. brasiliensis* infection in mice and purified gp43 lead to down-regulate properties of immature DCs (Ferreira et al., 2004).

### Spread *via* Transmigration of Infected Phagocytes

The Trojan horse-like mechanism was described for other fungal infections and well-studied in *Cryptococcus*, explaining the mechanism of cryptococcal brain invasion (Shi and Mody, 2016). As well as *Paracoccidioides*, cryptococcal infection begins in the lung and experimental evidence showed that host phagocytes play a role in subsequent dissemination. This transmigration model contributes significantly to fungal barrier crossing and *Cryptococcus*-containing phagocytes can cross the blood-brain barrier *via* transendothelial pores (Santiago-Tirado et al., 2017; Casadevall et al., 2018). Three mechanisms have been proposed for pathogens to cross the blood-brain barrier: transcellular migration, paracellular migration and/or by means of infected phagocytes (Trojan horse model), proliferating and causing grave illness (Shi and Mody, 2016). Evidence for this model were showed using mice infected with macrophages containing ingested cryptococcal cells (Charlier et al., 2009). Although Trojan horse-like mechanism has been more studied in *Cryptococcus*, and its glucuronoxylomannan capsule plays an important role in the inhibition of phagocytosis, *Paracoccidioides* deploys numerous effectives abilities to persist and also multiply inside phagocytes as a facultative intracellular pathogen (**Figure 1**). Therefore, access to this mechanism is feasible by *Paracoccidioides*, and dissemination to other organs/systems could occur (Brummer et al., 1989; Moscardi-Bacchi et al., 1994).

Although alveolar macrophages have well-defined immunoregulatory functions, these cells are generally considered as restricted to the alveoli. It was demonstrated that murine alveolar macrophages constitutively migrate from lung to the lung draining lymph nodes and that following exposure to bacteria, they rapidly transport bacteria to this site. Alveolar macrophages, such as DC, appear responsible for the earliest delivery of these bacteria to secondary lymphoid tissue. The identification of this transport suggests an important role for macrophages in the transport of invading pathogens to lymphoid organs (Kirby et al., 2009).

Non-lytic exocytosis for yeast infecting phagocytes where demonstrated. Viable yeast cells can come out of the macrophages without phagocytes lysis (Alvarez and Casadevall, 2006). Like other yeasts, *Paracoccidioides* could escape from intracellular confines of mammalian macrophages to continue propagation and, possibly, dissemination. Also *Candida albicans* spread *via* phagocyte-dependent mechanism. Using *in vitro* and zebrafish disease models, how neutrophils and macrophages can be vehicles for dissemination have been demonstrated. *Candida* *albicans* survive within macrophages and can be released far from the site of infection through non-lytic exocytosis. The intracellular viable yeast is able to get into the bloodstream and use blood flow to transmigrate to other tissues (Scherer et al., 2020).

In *Paracoccidioides* little is known about which pathways this fungus activates to escape from the monocyte-phagocyte system. Murine animal models are considered the gold standard for *in vivo* studies to simulate the fungal infection (De Oliveira et al., 2015). The migration of lung DCs to the lymph nodes and also lung DCs phagocyting *P. brasiliensis* yeast *in vivo* were demonstrated (Ferreira et al., 2007).

After *P. brasiliensis* infection, an increase in DCs expression of the chemokine receptors CCR7, CD103, and MHC-II occurs, enabling DCs migration from the infection site to the secondary lymphoid after interacting with the fungus. This fact indicate that *Paracoccidioides* induce migration of DCs. Animal model showed bone marrow-derived DCs stimulated by *P. brasiliensis* can migrate to the lymph nodes and activate a T-cell response. Even more, it was demonstrated *in vivo* that DCs migrate and transport the yeast parasitic form of the fungus to lymph nodes (Silvana dos Santos et al., 2011). This strategy allows *Paracoccidioides* to leave the lung and to penetrate other tissues protected by the phagocytic cells. Lung DCs could act as Trojan horses for this fungus.

## Discussion

*Paracoccidioides*-phagocytic cells interaction comprise a complex transcriptional and translational plan including a powerful antioxidant defense system. The host is under pressure to develop resistance while the parasite tries to tolerate, adapt to this new biotrophic lifestyle and overcome host environmental stressors and reach to subsist.

The recognition of the fungal cells by the capable host immune system trigger a large number of processes to control these organisms, but not only the immune responses pattern determines the progression of the disease and the clinical outcome. Despite the efficient host fighting and even when it has already been engulfed by phagocytes, we reviewed in this article the amazing set of tools and strategies exposed by *Paracoccidioides* to stay alive.

These pathogenic abilities allow not only their survival but the possibility of gain access to other tissues *via* transmigration of infected phagocytes. In this process, *Paracoccidioides* also causes phagocytes to play a dual role, they can contain the PCM or be instrumental to disseminate the infection. This mechanism, which actually includes a spectrum of strategies increases the virulence of this dimorphic fungus.

The Trojan horse mechanism represents a striking demonstration of the admirable adaptability of the yeast-like pathogenic form of *Paracoccidioides* to adverse conditions, as an accidental fact in the life cycle of this environmental fungi trying to survive after inhalation.

Nowadays, we understand better about how this fungus spreads throughout a host. However, although PCM poses a significant clinical risk, we still understand little about what roles plays the host in limiting or enabling its dissemination. The possibility of occurrence probably is not only related to the patient’s immune status, but on a multiplicity of factors including sex, age, lifestyle, its genetic background, and also the inhaled fungal load depending on the environmental context, among others.

## Author Contributions

The author confirms being the sole contributor of this work and has approved it for publication.

## Conflict of Interest

The author declares that the research was conducted in the absence of any commercial or financial relationships that could be construed as a potential conflict of interest.
